# Hypereosinophilic syndromes

**DOI:** 10.1186/1750-1172-2-37

**Published:** 2007-09-11

**Authors:** Florence E Roufosse, Michel Goldman, Elie Cogan

**Affiliations:** 1 Institute for Medical Immunology, Université Libre de Bruxelles, Gosselies, Belgium; 2 Department of Internal Medicine, Erasme Hospital, Université Libre de Bruxelles, Brussels, Belgium

## Abstract

Hypereosinophilic syndromes (HES) constitute a rare and heterogeneous group of disorders, defined as persistent and marked blood eosinophilia (> 1.5 × 10^9^/L for more than six consecutive months) associated with evidence of eosinophil-induced organ damage, where other causes of hypereosinophilia such as allergic, parasitic, and malignant disorders have been excluded. Prevalence is unknown. HES occur most frequently in young to middle-aged patients, but may concern any age group. Male predominance (4–9:1 ratio) has been reported in historic series but this is likely to reflect the quasi-exclusive male distribution of a sporadic hematopoietic stem cell mutation found in a recently characterized disease variant. Target-organ damage mediated by eosinophils is highly variable among patients, with involvement of skin, heart, lungs, and central and peripheral nervous systems in more than 50% of cases. Other frequently observed complications include hepato- and/or splenomegaly, eosinophilic gastroenteritis, and coagulation disorders. Recent advances in underlying pathogenesis have established that hypereosinophilia may be due either to primitive involvement of myeloid cells, essentially due to occurrence of an interstitial chromosomal deletion on 4q12 leading to creation of the *FIP1L1-PDGFRA *fusion gene (F/P^+ ^variant), or to increased interleukin (IL)-5 production by a clonally expanded T cell population (lymphocytic variant), most frequently characterized by a CD3^-^CD4^+ ^phenotype. Diagnosis of HES relies on observation of persistent and marked hypereosinophilia responsible for target-organ damage, and exclusion of underlying causes of hypereosinophilia, including allergic and parasitic disorders, solid and hematological malignancies, Churg-Strauss disease, and HTLV infection. Once these criteria are fulfilled, further testing for eventual pathogenic classification is warranted using appropriate cytogenetic and functional approaches. Therapeutic management should be adjusted to disease severity and eventual detection of pathogenic variants. For F/P^+ ^patients, imatinib has undisputedly become first line therapy. For others, corticosteroids are generally administered initially, followed by agents such as hydroxycarbamide, interferon-alpha, and imatinib, for corticosteroid-resistant cases, as well as for corticosteroid-sparing purposes. Recent data suggest that mepolizumab, an anti-IL-5 antibody, is an effective corticosteroid-sparing agent for F/P-negative patients. Prognosis has improved significantly since definition of HES, and currently depends on development of irreversible heart failure, as well as eventual malignant transformation of myeloid or lymphoid cells.

## Disease name and synonyms

### Hypereosinophilic syndromes (HES)

In this article, we have chosen to adopt the classification scheme proposed by Klion *et al*., on behalf of the Hypereosinophilic Syndromes Working Group that reached a consensus in Bern in 2005 [1]. Under the umbrella diagnosis of "Hypereosinophilic Syndromes", various disease presentations and pathogenic variants are divided in subgroups (see bullets below). This approach offers the advantage of providing physicians with the full array of differential diagnosis, once underlying diseases (parasitic, allergic, neoplastic *etc*.) classically known to be associated with hypereosinophilia are excluded by thorough patient evaluation. It provides a basis for developing a diagnostic and therapeutic management algorithm of chronic eosinophil-mediated disease.

The following terms are currently used in medical literature to qualify different patient subsets among those fulfilling the diagnostic criteria of HES:

• FIP1L1-PDGFRA(F/P)-associated HES, or F/P^+ ^HES, for patients with clonal hypereosinophilia due to a sporadic hematopoiëtic stem cell chromosomal rearrangement resulting in fusion of two genes (*FIP1L1 *and *PDGFRA*) on 4q12; these patients are more appropriately classified as F/P^+ ^chronic eosinophilic leukemia.

• Chronic eosinophilic leukemia (CEL), for patients in whom clonality of eosinophils has been demonstrated (including those with the FIP1L1-PDGFRA fusion), or who present increased blasts; occasional reports indicate that some patients with CEL progress towards acute myeloid or eosinophilic leukemia.

• Lymphocytic-HES (L-HES), for patients with chronic reactive (polyclonal) hypereosinophilia secondary to IL-5 over-production by T cells.

• Myeloproliferative-HES (M-HES) may be used for patients with an array of clinical and biological features suggesting the possible existence of an underlying myeloproliferative disorder associated with hypereosinophilia, although underlying molecular defects are not detected (including increased serum vitamin B12, hepato- and/or splenomegaly, anemia and/or thrombocytopenia, circulating myeloid precursors, dysplastic eosinophils, bone marrow hypercellularity with left shift in maturation, myelofibrosis, increased serum tryptase, and response to imatinib); this term is occasionally extended more largely to include patients with known molecular defects (*e.g*. F/P^+ ^CEL or other rare fusions involving the *PDGFRA *gene) as well.

• Idiopathic hypereosinophilic syndrome, for patients in whom underlying pathogenesis remains unknown.

• Organ-restricted eosinophilic disease, such as eosinophilic esophagitis, eosinophilic gastro-enteritis, eosinophilic dermatitis, eosinophilic pneumonia, Kimura's disease, eosinophilic fasciitis *etc*., for patients in whom a specific organ or tissue is the exclusive target of eosinophilic infiltration and damage.

Terms such as disseminated eosinophilic collagen disease or Löffler's fibroplastic endocarditis with eosinophilia were used previously to reflect end-organ damage (disseminated or cardiac, respectively) due to direct toxicity of circulating eosinophils in patients with persistent hypereosinophilia, but are currently out-dated.

## Definition and diagnostic criteria

The term "hypereosinophilic syndrome" was coined in 1968 to regroup patients with a number of closely related disorders, all characterized by chronically increased peripheral blood eosinophil levels, and organ damage related to eosinophilic infiltration [2]. A working definition of "idiopathic" HES was proposed by Chusid in 1975: sustained peripheral blood eosinophilia of unknown origin, exceeding 1.5 × 10^9^/L for more than six consecutive months, and responsible for the development of organ dysfunction and/or damage [3]. This definition was recently extended to include patients with clear-cut idiopathic hypereosinophilia responsible for end-organ damage, in whom treatment is initiated rapidly to prevent further damage, despite non-fulfillment of the duration criterion. In light of significant advances in understanding disease pathogenesis in specific patient sub-groups, it has recently been agreed that the term "idiopathic" be reserved for HES patients in whom pathogenesis remains entirely unknown, while the larger umbrella "hypereosinophilic syndrome(s)" be used once again to cover the heterogeneous disease group characterized by marked hypereosinophilia accompanied by eosinophilic infiltration of tissues, encompassing HES variants with well-characterized pathogenic mechanisms, idiopathic HES, familial HES, and organ-specific eosinophil-mediated disease (eosinophilic gastro-enteritis, eosinophilic pneumonia ...) (see ref [1] for a recent classification of these eosinophil-mediated disorders).

## Epidemiology

HES is a rare and under-diagnosed disorder, making it difficult to estimate overall prevalence. To date, there are no published data on epidemiology of the disease, but an estimate based on annual hospital discharges (patients with hypereosinophilia and pertinent co-morbidity) in the United States, and on United States claims database for capturing out-patient management, has indicated that HES could represent approximately a third of chronic myelogenous leukemia (CML) patients [4]. An improved approach to HES diagnosis in routine practice and creation of patient registries are essential steps before a reliable estimation of true prevalence can be made. HES predominantly affects males, with an estimated male to female ratio ranging between 4 and 9 to 1 [5]. This is partially explained by the overwhelming majority of males affected by the F/P^+ ^disease variant, characterized by occurrence of a sporadic hematopoietic stem cell mutation involving the *PDGFRA *gene. Disease tends to occur in patients aged from 20 to 50, but all age groups may be concerned [5].

## Etiology

Eosinophils belong to the myeloid lineage, and differentiate from myeloid progenitors (GEMM-CFU) in bone marrow. Among the three cytokines that act as eosinophil growth factors and apoptosis inhibitors, *i.e*. granulocyte-macrophage colony stimulating factor (GM-CSF), IL-3, and IL-5, only the latter displays specificity for eosinophils [6]. The major source of this eosinophil-specific cytokine is represented by so-called "type 2" helper T cells [7]. Recent studies based on material from patients fulfilling Chusid's HES diagnostic criteria have shown that two distinct underlying mechanisms may lead to chronic unexplained hypereosinophilia in HES patient subgroups: occurrence of a sporadic hematopoïetic stem cell mutation, leading to primitive clonal expansion of cells belonging to the myeloid lineage with preferential eosinophilic differentiation (as such, hypereosinophilia belongs to the group of chronic myeloproliferative disorders), or overproduction of eosinophilopoietic cytokine(s) by an activated population of T cells (in the "lymphocytic variant" of HES, or L-HES) ([for a review see [8]]).

Until recently, it was difficult to prove eosinophil clonality and/or to identify a mutation in patients with features of myeloproliferative disease, and for whom investigators were convinced an underlying stem cell disorder was present. In rare cases, some authors were able to show eosinophil clonality if overt chromosomal abnormalities were present, and for female patients in whom skewed methylation patterns of X-linked genes could be demonstrated in purified eosinophils [9]. Recently, a cryptic cytogenetic abnormality (which is invisible on routine karyotypes) has been identified in eosinophils from a significant proportion of HES patients, establishing the existence of a clonal myeloproliferative disorder [10]. Briefly, an interstitial deletion on chromosome 4q12 results in fusion of two genes, *FIP1L1 *and *PDGFRA*. The new fusion gene encodes a FIP1LI-PDGFRA (F/P) protein displaying constitutive tyrosine kinase activity, whose role in disease induction has been confirmed by its disappearance in patients successfully treated with the tyrosine kinase inhibitor, imatinib. Furthermore, the F/P fusion is implicated in malignant transformation of eosinophils/myeloid cells, as indicated by its ability to render a murine hematopoietic cell line independent of growth factors *in vitro *following transfection [10], and by its presence in a cell line derived from a patient with acute eosinophilic leukemia [10,11]. Although eosinophils are by far the predominant leukocyte in blood and tissues from patients with the F/P fusion, this clonal abnormality has been demonstrated in a number of other cell lineages [12], including mast cells, which display morphological changes and which may be the source of increased serum tryptase levels observed in these patients [13]. The proportion of patients initially fulfilling diagnostic criteria for HES in whom the F/P mutation is detected varies among reports, ranging between 17% [14] and 56% [10]. This variability reflects differences in patient referral to medical sub-specialties, depending on predominant clinical manifestations and complications of hypereosinophilia. Experts currently agree that patients with F/P-associated disease should be classified as chronic eosinophilic leukemia (CEL); however, other terms used in medical literature include F/P-associated HES or disease, F/P^+ ^HES, or M-HES. Whether this well-defined patient subgroup should remain under the umbrella diagnosis of HES or not remains a controversial issue. From a practical stand-point, we and others consider that separating disease variants before further progress is made in understanding pathogenesis of persistent hypereosinophilia, will only confuse physicians. F/P^+ ^CEL is therefore included in the overall HES disease spectrum, which in no way interferes with the formulation of special recommendations regarding diagnosis and management [15].

In L-HES, overproduction of eosinophil growth factors by T cells leads to increased cycling, differentiation and maturation of eosinophil precursors, as well as prolonged survival of eosinophils in the periphery, resulting in non-clonal hypereosinophilia. Interleukin-5-producing T cell subsets have been described in blood of approximately 35 patients with HES, and a rough estimate would be that at most one fourth of HES patients present this variant [8]. The allegedly pathogenic T cells display an aberrant surface phenotype in all reported cases, and while CD3^-^CD4^+ ^cells represent the most frequently encountered subset in this setting, CD3^+^CD4^-^CD8^-^, CD4^+^CD7^- ^and other populations have also been reported [16]. In addition to IL-5, the CD3^-^CD4^+ ^cells produce other Th2 cytokines such as IL-4 and IL-13, as well as GM-CSF. Effects of the Th2 cytokines IL-4 and IL-13 on other cells account for associated biological features of L-HES. Indeed, B cell stimulation leads to increased IgE synthesis and polyclonal hypergammaglobulinemia; and effects on antigen presenting cells and/or epithelial cells lead to high-level production of thymus and activation-regulated chemokine (TARC), which is increased in serum from such patients [17]. Clonality of phenotypically aberrant T cells has been demonstrated in many cases, by analysis of *TCR *gene rearrangement patterns. Clonal cytogenetic abnormalities, including 16q breakage, partial 6q or 10p deletions [18], and trisomy 7 have been reported. Although the clonal T cells are initially benign, several authors have reported progression towards full-blown T cell lymphoma, indicating that this initially benign lymphoproliferative disorder potentially represents a pre-malignant state.

Finally, a good half of patients with HES remain unclassified, and present truly "idiopathic" hypereosinophilia. Among these, some present features of myeloproliferative disease similar to those encountered in F/P^+ ^individuals, whereas others appear to have more of an "immuno-allergic" disease suggesting possible involvement of T cells. Further investigation of eosinophils and T cells in these idiopathic cases, will very likely lead to identification of novel molecular mechanisms ultimately leading to hypereosinophilia.

## Clinical description

### Clinical manifestations

The clinical manifestations of HES are variable from one patient to another, depending on target-organ infiltration by eosinophils. Organ damage and/or dysfunction develops as a result of eosinophil release of a number of cytotoxic substances, including highly cationic molecules such as eosinophil cationic protein (ECP) and major basic protein (MBP), the ribonuclease eosinophil derived neurotoxin (EDN), oxydating molecules such as eosinophil peroxidase (EPO) and free oxygen radicals, and enzymes such as elastase and collagenase [6]. Eosinophils are also able to produce lipid mediators, such as leukotrienes and prostaglandins, which contribute to clinical complications through effects on vascular and bronchial smooth muscle tone. Finally, their ability to secrete cytokines (including pro-inflammatory, Th1 and Th2 cytokines) and chemokines makes them more sophisticated participants in the immune response than was once thought [19]. Eosinophilic inflammation is associated with a pro-fibrotic environment, as release of TGF-**β **by activated eosinophils leads to increased collagen synthesis and extracellular matrix deposition. Other mediators of eosinophil-induced tissue remodeling are the subject of research efforts.

Although virtually any tissue or organ can be affected in HES, clinical complications arise most frequently (in more than 50% of patients) in the skin, heart, lungs, and nervous system [5]:

• **Cutaneous manifestations **are common and non-specific, and generally consist either in angioedematous and urticarial lesions, or erythematous, pruritic papules and nodules resembling eczema. In patients with L-HES, such lesions are often the predominant clinical complication of hypereosinophilia [16,20]. Typical features that compose Gleich's syndrome, or episodic angioedema with eosinophilia, have been reported in some patients with L-HES [20-22]. Mucosal ulcerations are possible, especially in patients with F/P-associated HES [13].

• **Cardiac involvement **generally evolves in three stages. The early necrotic stage which involves the endo-myocardium is often asymptomatic, but may present as acute heart failure in rare instances. This stage is followed by a thrombotic stage in which thrombi develop along the damaged endocardium in the cardiac chambers and may detach, causing peripheral emboli. In the final fibrotic stage, endomyocardial fibrosis results in irreversible restrictive cardiomyopathy, and damage to atrioventricular valves may lead to more acute presentations with congestive heart failure [23]. Endomyocardial fibrosis is the most pre-occupying non-malignant complication of F/P-associated disease, as it is generally irreversible [24]. Chest discomfort, cough, dyspnea or orthopnea, edema of the lower extremities are typical symptoms reflecting cardiac involvement, and patients may develop arrhythmias.

• **Neurological manifestations **may involve both central (diffuse encephalopathy) and peripheral (polyneuropathy) nervous systems. Diffuse encephalopathy presents as altered behavior and cognitive function, confusion, and memory loss. Peripheral neuropathies present as symmetric or asymmetric sensory changes, pure motor deficits, or mixed sensory and motor complaints. Stroke or transient ischemic episodes may occur following peripheral embolism of intracardiac thrombi. Thrombosis of intracranial veins (longitudinal and/or lateral sinus) is a possible complication of the pro-coagulant state associated with persistent hypereosinophilia in some patients. To date, no patients with L-HES have been reported to present neurological complications, in contrast to F/P^+ ^and idiopathic patients.

• **Lung involvement **can range from chronic dry cough and/or bronchial hyperreactivity in absence of radiological abnormalities, to restrictive disease with pulmonary infiltrates. The latter may occur more frequently in patients with F/P-associated disease [13]. In rare instances, development of acute respiratory distress syndrome has been reported. Pulmonary fibrosis may develop after prolonged disease.

• **Hematological manifestations **include anemia, thrombocytopenia, hepatomegaly and splenomegaly, all of which are more typically encountered in association with the F/P fusion [13,25]. Mild lymphadenopathy is occasionally observed in patients with L-HES; and appearance or modification in size of lymph nodes should arouse suspicion of T cell lymphoma.

• **Coagulation disorders **may be observed in HES, and it is believed that chronic hypereosinophilia may cause damage to the endovascular surface, accounting for peripheral vasculopathy, and may also activate coagulation directly. Special attention should be paid to the presence of nail-fold splint hemorrhages, as peripheral thromboembolic phenomena may result in occlusion of terminal vessels in some patients [26] (*e.g*. digital necrosis).

• **Gastrointestinal symptoms **may include abdominal pain, diarrhea, nausea and vomiting. Eosinophilic gastritis, enterocolitis, or colitis may be present, and the latter may be associated with ascitis if eosinophilic infiltrates involve deeper layers of the intestinal wall. Such digestive complications are observed in all forms of HES, including those with unknown pathogenesis.

• **Constitutional symptoms **include weakness, fatigue, anorexia, fever, night sweats, weight loss, arthralgia and myalgia.

### Biological findings

HES patients are also heterogeneous with regard to associated biological features. Total leukocyte counts can be normal or increased, and absolute eosinophil numbers are often well over the defined 1.5 × 10^9^/L. A number of hematological abnormalities may be observed in peripheral blood, especially in F/P^+ ^disease, including presence of myelocytes, thrombocytopenia or thrombocytosis, and anemia. Marked increases in serum vitamin B12 levels are observed in patients with F/P^+ ^disease, and are considered as indicative of M-HES (*i.e*. probable myeloproliferative disorder due to yet unidentified molecular defects), together with the above-mentioned hematological abnormalities and organomegaly [13,25]. High serum immunoglobulin E (IgE) levels and polyclonal hypergammaglobulinemia are more typical of, but not restricted to, L-HES [20].

### Disease course and outcome

Chusid's initial paper and the following published series clearly illustrated the great clinical heterogeneity and highly variable prognosis of HES, ranging from paucisymptomatic disease requiring no treatment and associated with prolonged survival, to rapidly fatal disease course due to the sudden development of congestive heart failure or acute leukemia. With the recent distinction of myelo- *versus *lympho-proliferative variants, subgroups of patients with more homogenous clinical profiles can be defined. Thus, patients with F/P-associated disease often present heart involvement and mucosal ulcers, and are overall more likely to develop disease-related morbidity and mortality [13], whereas patients with L-HES often present predominant cutaneous manifestations, while their cardiovascular system is often spared [20]. As for long-term hematological outcome, patients with F/P-associated disease are at risk for developing acute leukemia (eosinophilic or myeloid) [25], at times shortly after diagnosis of HES, while patients with L-HES are at risk of developing T cell lymphoma, generally after many years of indolent pre-malignant disease [16,20,27,28].

Although prognosis of HES was very poor when the syndrome was first described (88% mortality at 3 years, see [3]), it has steadily and significantly improved over time for a number of reasons including earlier detection of complications, better surgical management of cardiac and valvular disease, and use of a wider spectrum of therapeutic molecules for controlling hypereosinophilia. Currently, prognosis depends on two major aspects of disease: heart involvement and the increased likelihood of developing hematological malignancies. It is likely that current and future therapeutic strategies taking pathogenic disease variants into account will further improve outlooks for patients with HES in the near future.

## Diagnostic methods

Initial work-up of HES requires rigorous step-wise progression through a diagnostic algorithm. When confronted with a new hypereosinophilic patient, the physician must first exclude all diseases known to be associated with hypereosinophilia before considering diagnosis of HES. Secondly, complications of chronic hypereosinophilia *per se *must be assessed. And thirdly, an attempt should be made to determine whether hypereosinophilia arises in the context of a primitive myeloid *versus *lymphoid disorder, *i.e*. to classify patients with regard to disease variant. The diagnostic procedures (including patient history and examination, and informative laboratory and imaging studies) to exclude underlying diseases associated with hypereosinophilia (see differential diagnosis) will not be detailed in this review.

Organ involvement in HES is highly variable from one patient to another with regard to its nature and severity. In a given patient, however, disease flares will often be accompanied by similar clinical manifestations. Evaluation of complications should obviously be guided by clinical assessment and physical examination, with special emphasis on the systems known to be targeted by eosinophils: cutaneous, cardiovascular, nervous, respiratory and digestive systems. Minimal investigations when diagnosis of HES is established include an electrocardiogram, echocardiogram, pulmonary function test, and a chest X-ray. Other imaging studies and/or biopsies should be performed based on clinical findings.

Classification of HES patients remains difficult in a routine setting, and requires referral of blood and/or marrow samples to qualified laboratories for optimal evaluation. Clinically, predominant cutaneous manifestations in absence of heart involvement, associated with serum hyperIgE and/or polyclonal hypergammaglobulinemia should arouse suspicion of L-HES [20]. Likewise, splenomegaly, heart involvement, mucosal ulcerations, increased vitamin B12 levels, anemia and/or thrombocytopenia, and presence of myeloid precursors in peripheral blood are indicative of F/P-associated HES [13]. However, none of these features are entirely specific for a disease variant, and further testing is warranted for appropriate categorization and treatment of patients. At the present time, standard care demands that the three following tests be performed on peripheral blood and/or bone marrow for all patients: search for the F/P fusion gene using RT-PCR (reverse transcription polymerase chain reaction, preferably nested for increased sensitivity) and/or FISH (fluorescent *in situ *hybridisation) for the CHIC2 locus (absence of this locus is considered a surrogate marker for presence of the F/P fusion [29]), lymphocyte phenotyping, and analysis of T cell receptor (*TCR*) gene rearrangement patterns. Indeed, presence of the F/P fusion clearly identifies patients with a distinct disease variant that responds dramatically to treatment with the tyrosine kinase inhibitor imatinib (see below). On the other hand, lymphocyte phenotyping in search of a phenotypically aberrant T cell subset (principally CD3^-^CD4^+^, CD3^+^CD4^-^CD8^-^, and CD4^+^CD7^-^), and analysis of *TCR *gene rearrangement patterns using both Southern Blot and PCR amplification for *TCR *gene variable regions, in search of T cell clonality, are generally sufficient to detect patients with an underlying T cell disorder.

However, despite adherence to the preceding guidelines, a number of patients will remain unclassified. Additional investigations in a research setting that may help distinguish myeloid and lymphoid disorders include: analysis of cytokine profiles of T cell subsets including eosinophilopoietic (IL-3 and GM-CSF) and/or type 2 (IL-4, IL-5, IL-13) cytokines [16,20], measurement of serum tryptase [13] and TARC levels [17], and cytogenetic analysis focusing on imatinib target tyrosine kinases [30]. There is a general consensus among experts in the field that serum or plasma IL-5 levels are of little value for classification, as activated eosinophils themselves are able to produce and secrete this cytokine.

## Differential diagnosis

When thorough evaluation of a patient with persistent hypereosinophilia fails to reveal an underlying disease known to be associated with increased eosinophil levels, diagnosis of "HES" can be considered. In the majority of cases, diagnostic work-up will ultimately lead to diagnosis of an allergic disorder or parasitosis. Other less frequent causes of hypereosinophilia include hematological malignancies such as CMML-Eos, Hodgkin's disease and cutaneous T cell lymphoma, solid tumors involving the lungs and colon, systemic vasculitides such as Churg-Strauss syndrome, connective tissue disease such as rheumatoid arthritis, infectious disease such as scabies, HIV (human immunodeficiency virus), HTLV (human T cell lymphotropic virus), and allergic bronchopulmonary aspergillosis, skin disease such as psoriasis, endocrine disease such as adrenal insufficiency, metabolic disease such as cholesterol embolism, and possible toxicity such as in the historic eosinophilia-myalgia syndrome.

Some hypereosinophilic patients in whom extensive investigations are negative for underlying diseases present a characteristic disease pattern in which a given tissue or organ is specifically targeted by eosinophils. Many of these idiopathic tissue-specific eosinophil-mediated disorders have been isolated from HES as distinct diseases, given their tendency to recur only in the initially affected organ, in absence of other complications of hypereosinophilia. Examples include eosinophilic gastroenteritis, chronic eosinophilic pneumonia or Carrington's disease, eosinophilic fasciitis, eosinophilic cellulitis or Wells' syndrome, episodic angioedema with eosinophilia or Gleich's disease, and Kimura's disease. However, whether a given patient with initially organ-restricted disease will eventually develop multi-system involvement is impossible to determine at presentation. For this reason, these disease presentations have been included in an overlap category under the umbrella of HES in a recent classification [1]. Also, as knowledge on pathogenesis accumulates, underlying disease mechanisms will over-ride tissue-specific classifications, as is already the case for patients with episodic angioedema, some of which present a CD3^-^CD4^+ ^T cell subset indistinguishable from that found in L-HES patients with isolated eosinophilic dermatitis (or eczema).

## Treatment

HES are chronic long-standing disorders that develop in young to middle-aged patients, meaning that therapeutic options should take long-term toxicity into consideration. Corticosteroids and hydroxycarbamide have been the cornerstones of management since definition of the syndrome in 1975 [5], and interferon alpha (IFN-**α**) was introduced in the early 1990s on the basis of several encouraging studies [31]. A strong need for new therapeutic alternatives that specifically target molecular mechanisms underlying hypereosinophilia, while causing as little unintentional damage as possible, has been voiced by physicians, given the poor tolerance and numerous side effects of these compounds. With the recent description of the F/P fusion with constitutive tyrosine kinase activity in a subgroup of patients, and demonstration of increased IL-5 production by abnormal T cells in others, therapeutic perspectives have radically changed in the past few years.

### F/P-associated HES

As mentioned in the paragraph on diagnosis of disease variants, optimal patient management relies on early testing for the F/P fusion gene. There is general consensus among experts in the field that patients in whom this chromosomal rearrangement is detected should be treated with the tyrosine kinase inhibitor imatinib (Gleevec^®^) as first line therapy [1]. A number of clinical studies showing the striking rapidity and potency of its effects in this selected patient population have been published, and to date, no case of primary resistance to the molecule has been reported [10,11,24,32]. Response to therapy in terms of eosinophil levels occurs within days in most cases, and many clinical manifestations can be reversed (including dermatitis, mucosal ulcers, restrictive lung disease, gastrointestinal involvement, central nervous system manifestations, certain cardiac manifestations, anemia, thrombocytopenia, and splenomegaly). The dose required to induce and maintain remission is generally lower (100 mg/day) than for patients with CML (≥ 400 mg). Influence of imatinib on clinical manifestations related to HES heart involvement are variable, and some authors have reported that endomyocardial fibrosis and related loss of function are not reversible [24,25]. Reversal of bone marrow pathology and molecular remission, a major endpoint when dealing with disease mediated by constitutively activated tyrosine kinases, can be achieved in most patients with the F/P fusion gene [24,33]. Overall, imatinib used at doses effective for HES is well tolerated, as most side effects including edema, muscle pain and fatigue, are dose-dependent. However, there is some concern regarding adverse effects of imatinib on heart function. Firstly, a few F/P^+ ^HES patients have developed severe congestive heart failure within days after initiation of therapy, and this was felt to be due to massive liberation of toxic eosinophil contents following imatinib-induced eosinophil death [34,35]. Rapid administration of corticosteroids has proven effective in handling this preoccupying complication [34]. Authors have suggested that heart function be monitored closely prior to and at the beginning of treatment, and serial measurements of cardiac troponin T be performed to predict this potentially fatal adverse event. Patients with increased levels prior to therapy should be treated preventively with corticosteroids a few days before initiating imatinib. Secondly, a recent study investigating cardiomyocytes from CML patients treated with imatinib (generally used at higher doses than for HES patients) and subsequently developing left ventricular dysfunction (after a mean of 7.2 months of treatment), has shown presence of membrane whorls and pleomorphic mitochondria with effaced cristae [36]. The mechanisms underlying imatinib-induced myocyte toxicity were investigated in mice, in which mitochondria were also identified as the chief target of this compound. Energy rundown due to mitochondrial dysfunction was shown to be responsible for cardiotoxicity.

Relapse of hypereosinophilia during treatment with imatinib has been reported in two F/P^+ ^patients, and was associated with appearance of a T674I point mutation in the ATP-binding site of the PDGFRA moiety, similar to the T315I mutation observed in patients with CML that become refractory to treatment [10,37]. It has been recommended that the dose of imatinib used for patients with F/P^+ ^HES should be adjusted to ensure molecular remission, in order to decrease the risk of acquired resistance to treatment [1]. Several alternative tyrosine kinase inhibitors have been tested *in vitro *and *in vivo *(murine model of F/P-associated disease) for effects on F/P activity. One molecule, nilotinib (AMN107), is able to inhibit kinase activity of wild-type F/P [38]. Two other compounds, PKC412 which is structurally unrelated to imatinib [39], and sorafenib [40], are able to inhibit kinase activity of both wild-type F/P and its imatinib -resistant T674I mutant form.

Although imatinib has clearly become first-line therapy for patients with F/P-associated disease, overall follow-up of treated patients is short, and a number of questions remain unanswered. Namely, it is currently unclear whether imatinib can be curative for HES, through permanent eradication of the leukemic F/P^+ ^clone. Several reports have shown that interruption of imatinib in F/P^+ ^patients in molecular remission, is followed by recurrence of the molecular defect within months [33,41].

### F/P-negative HES

To date, no general consensus has been reached on the ideal treatment algorithm for HES patients without the F/P fusion. In general, corticosteroids are administered as first line therapy, starting with a dose of 1 mg/kg/d or 60 mg prednisone. If a response is observed, prednisone is carefully tapered to the lowest possible dose that maintains eosinophil counts and/or clinical manifestations under control, defining the level of corticosteroid-dependency. Depending on the dose of corticosteroids required and on patient tolerance, physicians frequently attempt to further reduce the dose by introducing a corticosteroid-sparing agent. If no response to corticosteroids is observed (*i.e*. corticosteroid-resistance), second-line therapy is warranted.

Compounds that are used for corticosteroid-sparing and for second-line purposes include hydroxycarbamide, IFN-**α **and imatinib. Recent studies indicate that monoclonal anti-IL-5 antibodies may represent an interesting therapeutic alternative. Other compounds such as ciclosporin, vincristine, and anti-CD52 antibodies (alemtuzumab) have proven useful in some patients. Available data on administration of cytotoxic molecules such as cyclophosphamide, methotrexate, busulfan, and chlorambucil, are not that encouraging and there are currently no recommendations in favor of their use in HES. Choice of which agent to use in a given patient is up to the clinician, and the following paragraphs review briefly the characteristics of available alternatives.

Hydroxycarbamide has been used extensively for treating HES, generally at doses between 1 and 2 g/d [1,5]. The effect of hydroxycarbamide on eosinophilia is central, meaning that reduction of eosinophil levels is not to be expected before two weeks after initiation of therapy. Adverse events are frequently observed, including hematological toxicity and gastrointestinal intolerance. Published reports on successful treatment of HES with hydroxycarbamide lack detailed clinical and diagnostic information that would allow speculations on which disease variants could benefit from therapy. Theoretically, this compound would appear more useful for treating patients with myeloproliferative features; however, it effectively lowered eosinophil levels in one patient with a CD3^-^CD4^+ ^clone. Some investigators have combined hydroxycarbamide and IFN-**α **in order to lower individual doses of each compound, thereby increasing overall tolerance [42].

Interferon-**α **has also been used successfully for management of HES [31]. Although highly variable dosing regimens have been used, it appears that doses of 1–2 million units/d are often sufficient to control eosinophil levels. It may take weeks before a response is observed, meaning that it may take months before a stable effective dose is reached. Common side effects include flu-like symptoms that tend to improve over time, depression, fatigue, and increased liver transaminases. Many IFN-**α **responders reported in the literature present a number of features suggestive of myeloproliferative disease, but these reports pre-date description of F/P-associated disease and L-HES. A corticosteroid-sparing effect of IFN-**α **has been observed in two of our patients with CD3^-^CD4^+ ^clones, which could be related to inhibition of IL-5 production [43] and anti-proliferative effects. However, these encouraging results are challenged by the observation that IFN-**α **prolongs survival of clonal CD3^-^CD4^+ ^cells *in vitro *by inhibiting spontaneous apoptosis, and may therefore provide these cells with a selective advantage [44]. Given the malignant potential of aberrant T cells associated with L-HES, we would recommend avoidance of IFN-**α **as monotherapy in this setting, and prefer combining with corticosteroids.

In F/P-negative HES patients, the place of imatinib among therapeutic options has not yet been defined. Several investigators have reported responses to imatinib in a variable proportion of such patients, suggesting that an unidentified cytogenetic rearrangement leads to acquisition of imatinib-sensitive autonomous tyrosine kinase activity. To date, there is no data available on potential biomarkers that would help identify F/P-negative patients with imatinib-sensitive disease. A short course of imatinib 400 mg daily could be proposed to patients with clinical and biological findings typically encountered in myeloproliferative disease (see those listed above for "M-HES") and those resistant to therapy with corticosteroids; rapid evidence of a hematological response would be encouraging for treatment prolongation. In a recent review of 94 published cases of HES treated with imatinib, it was suggested that presence of splenomegaly or lung disease could be associated with a higher probability (89% and 96% respectively) of complete hematological response to imatinib [45]. In contrast to patients with F/P-associated disease, response of F/P-negative patients to imatinib is variable, and may only be transient in some cases, or may require higher doses.

The recent development of humanized anti-IL-5 mAbs, designed to target eosinophils in allergic disorders by interfering with ligation of IL-5 to the **α **-chain of the IL-5R on their surface, has raised considerable interest among investigators dealing with HES [46]. There is strong scientific rationale for treatment of HES patients with anti-IL-5 mAb, given the specificity of this cytokine for the eosinophil lineage, and the assumption that tissue damage in HES is directly related to presence of activated eosinophils. Initially, two molecules were developed for intravenous use (mepolizumab by GlaxoSmithKline and formerly SCH55700 by Schering Plough) and tested in HES patients, some of whom were corticosteroid non-responders, in the setting of compassionate use programs. Encouraging results were reported, with a rapid decline of blood eosinophil counts shortly after administration in most patients [47-49]. This was associated with decreased eosinophil degranulation, reflected by reduced serum eosinophil cationic protein (ECP) levels [47]. In most cases, successful eosinophil depletion in blood was associated with improvement of a wide spectrum of clinical manifestations (including rash, angioedema, mucosal ulcers, myalgia, arthralgia, dysphagia, vomiting, nasal congestion and polyposis), correlating with significant reductions of eosinophil numbers in the skin and esophagus of patients with eosinophilic dermatitis [47] and severe eosinophilic esophagitis [48], respectively. Among patients under corticosteroids before treatment initiation, several could be tapered down or off after mepolizumab infusions, supporting its use as a corticosteroid-sparing agent in HES. Eosinophil depletion and clinical remission following administration of 750 mg mepolizumab appears to last weeks or even months in some cases, and two patients with eosinophilic dermatitis have experienced persistent remission (with follow-up of 17 months in one case) following treatment discontinuation [47]. These individual compassionate case studies have suggested that HES patients with various profiles could benefit from treatment with anti-IL-5, including corticosteroid-responders and non-responders, and patients with both myeloproliferative (including one patient in whom the F/P fusion gene was later demonstrated [49]) and possible T cell mediated disease [47].

Although SCH55700 is no longer available, mepolizumab has recently been administered to a large number of F/P-negative corticosteroid-responsive HES patients in the setting of an international multicentric, randomized, double-blind, placebo-controlled clinical trial (750 mg administered intravenously every 4 weeks) [50]. This study has shown that mepolizumab allows effective corticosteroid-sparing compared to placebo, while maintaining disease control, and is well tolerated. An open-label extension study is currently on-going, addressing the optimal dosing interval between mepolizumab infusions, as well as long-term side effects associated with treatment itself and prolonged eosinopenia. Hopefully, additional analysis will identify biomarkers predictive of response to therapy with anti-IL-5.

Although anti-IL-5 mAbs display an excellent safety profile with minimal if any reported side effects, some concern regarding rebound hypereosinophilia between infusions or after cessation of therapy has been raised. One study using SCH55700 at low doses (1 mg/kg) has shown that serum IL-5 levels actually increased during treatment, and this appeared not to be related to increased IL-5 production by peripheral blood leukocytes [49]; mechanisms underlying IL-5 over-production or decreased IL-5 clearance during therapy remain to be clarified. Whether a similar phenomenon occurs with mepolizumab, remains unknown, and will be evaluated in the setting of the above-mentioned clinical trials.

An interesting therapeutic target in patients with L-HES is the CD52 antigen, which is expressed both on T cells and eosinophils. In a recent report, alemtuzumab, a monoclonal anti-CD52 antibody, was shown to be effective treatment for a patient with a CD3^-^CD4^+ ^T cell subset, inducing rapid normalization of eosinophil levels and clinical remission [51].

Ciclosporin interferes with calcium-mediated intracellular signaling pathways and inhibits nuclear translocation of the transcription factor NF-AT, which is essential for a number of T cell functions including cytokine synthesis. Ciclosporin is classically used for treating auto-immune disorders, by targeting T cells. A few authors have reported successful use of ciclosporin as a corticosteroid-sparing agent in a limited number of HES patients [52]. Given the pathogenic role of T cells in L-HES, and dependence of CD3^-^CD4^+ ^T cells on IL-2 for proliferation and cytokine production [53], ciclosporin or other agents interfering with IL-2/IL-2R interactions, may theoretically be useful for this HES variant. This remains entirely to be assessed.

Vincristine is a cytotoxic agent that is rarely used for management of HES. It may prove useful for rapidly lowering eosinophilia in patients with extremely high eosinophil counts (> 100 × 10^9^/L), and has been proposed in some pediatric cases that are refractory to classical therapeutic regimens [1]. The recommended dose for adults is 1–2 mg intravenously.

Finally, HES patients that are refractory to classical therapy and who present progressive life-threatening end-organ damage may be candidates for allogeneic stem cell transplantation (SCT), whether they test positive for the F/P fusion or not. Obviously, this strategy requires careful thought given the inherent morbidity and mortality related to the procedure. Potential indications for SCT may include patients with F/P-associated disease who are intolerant to or no longer respond to imatinib [1], and patients initially presenting L-HES who develop peripheral T cell lymphoma, as eradication of malignant T cells is not easily achieved using classical chemotherapeutic regimens.

Overall, management of patients with HES has improved significantly, especially since the rapid initiation of imatinib in patients with F/P^+ ^disease. As for L-HES, there is now increased awareness that despite the clinically "benign" presentation with relative sparing of end-organs, patients should be closely monitored for development of T cell malignancy. However, the molecular picture of L-HES remains uncomplete and specific molecular targets for therapy have not been identified, meaning that in the meantime therapy relies largely on corticosteroids, with eventual addition of corticosteroid-sparing agents such as IFN-**α**. Interesting options for future investigation may include alemtuzumab, extracorporeal photopheresis, and efalizumab (J. Huss-Marp, personal observation).

## Unresolved questions

• Striking male predominance of F/P-associated hypereosinophilic disease.

• Identification of underlying hematological abnormality in patients in whom diagnostic work-up fails to demonstrate eosinophil clonality (for example F/P fusion gene) or T cell populations producing eosinophilopoietic cytokines (L-HES). This includes characterization of the underlying tyrosine kinase abnormality in patients responding to imatinib, and lacking the F/P mutation.

• Long-term effects of imatinib on natural disease course of F/P-associated disease (*i.e*. ability or not to prevent blastic transformation, occurrence of imatinib-resistance).

• Role of the CD3-negative phenotype of pathogenic T cells underlying L-HES in malignant progression towards lymphoma.

• Identification of T cell molecular targets in patients with L-HES, for development of specific disease-modifying therapeutic strategies.

## Abbreviations

cGVHD chronic graft versus host disease

CEL chronic eosinophilic leukemia

CMML-Eos chronic myelo-monocytic leukemia with eosinophilia

ECP eosinophil cationic protein

EPO eosinophil peroxydase

FIP1L1 FIP1 (gene derived from Saccharomyces Cervisiae) like 1

F/P FIP1L1-PDGFRA fusion

GM-CSF granulocyte macrophage colony stimulating factor

HES hypereosinophilic syndrome

HIV human immunodeficiency virus

HTLV human T cell lymphotropic virus

IFN-**α **interferon alpha

IL interleukin

MBP major basic protein

PCR polymerase chain reaction

PDGFRA platelet derived growth factor receptor alpha

TCR T cell receptor
